# Dehumanization in female victims of intimate partner violence

**DOI:** 10.5249/jivr.v15i1.1676

**Published:** 2023-01

**Authors:** Homa Shahbazi, Mansoureh Alsadat Sadeghi, Leili Panaghi

**Affiliations:** ^*a*^ Faculty of Education and Psychology, Shahid Beheshti University, Tehran, Iran.; ^*b*^ Department of Family Therapy, Family Research Institute, Shahid Beheshti University, Tehran, Iran.

**Keywords:** Dehumanization, Intimate partner violence, Domestic violence, Grounded theory

## Abstract

**Background:**

Dehumanization is a psychological construct meaning denying a person’s humanity. The present study has investigated the process of dehumanization in female victims of intimate partner violence using the grounded theory approach.

**Methods:**

For this purpose, 130 women in Tehran were selected through the convenience sampling method, and after completion of the Revised Conflict Tactics Scales (CTS2), 60 female victims were identified. In-depth interviews started with these women, and the data reached theoretical saturation by interviewing ten victims.

**Results:**

The data analysis shows the role of dehumanization in the experiences of female victims of intimate partner violence in the form of two models. The first model showed that from the victim’s perspective, dehumanization plays a crucial role in intimate partner violence. The second model showed that dehumanization was experienced by these women and is involved in developing strategies in their response to the violence. The combination of these two models showed that dehumanization and violence in the context of domestic violence have a reciprocal relationship, forming a cycle between cognitions, emotions, and negative behaviors between couples.

**Conclusions:**

Data analysis demonstrated that dehumanization might have a role in experiencing intimate partner violence and contribute to IPV recurrence.

## Introduction

Dehumanization is a psychological construct defined as denying an individual’s humanity.^1^ Based on Haslam’s^2^ dual model of dehumanization, there are two different forms of humanness, and denying each of them leads to the two corresponding forms of dehumanization: animalistic dehumanization, which involves denying uniquely human (UN) traits that distinguish humans from animals, and mechanistic dehumanization, which consists of denying human nature (HN) traits that distinguish human from objects and automatons. Over the past decade, most research in this field has emphasized intergroup violence (e.g., between ethnic groups).^3^ However, recent studies have shown that this phenomenon can be subtle and hidden in daily maltreatment.^2^

**Violence against women and dehumanization**

Domestic violence is a major public health issue, a violation of human rights, and a significant risk factor for developing mental and physical diseases.^4^ IPV is one of the most common forms of domestic violence and refers to any intentional abuse perpetrated by an intimate partner^5^ and can be in the form of physical, sexual, and psychological violence or a combination of them.^6^ Like other forms of violence, dehumanization can be involved in IPV.^7^ For example, dehumanization may devalue women, which allows any control and physical harm as in physical violence, direct or implied contempt of women and justifying controlling them as a part of men’s property as in psychological violence, and also allows relegating women to a sexual object, lifeless and lacking in humanity, which justifies sexual violence.^8^ Rudman and Mescher^9^ also showed that sexual harassment of women and raping is more probable in men who implicitly dehumanize women. Most studies on violence against women have only focused on sexual violence. Furthermore, although some research has been carried out on university students, there is very little scientific understanding of the role of dehumanization in violence against women in a broader context.

**Experiencing dehumanization**

Previous researchers have traditionally focused on the dehumanization of others^10,11^ rather than the viewpoints of the dehumanized individuals. In recent years, there has been an increasing interest in dehumanized individuals’ perspectives. Bastian and Haslam^12^ showed that people might perceive themselves as less than human when exposed to negative behaviors. Moreover, Bastian and Haslam^13^ found that people dehumanize themselves when they experience social exclusion. Experiencing violence and abuse can change the view of the affected individual toward self, others, and the world.^14^ Particularly, if this violence, rape, or sexual harassment occurs in an intimate relationship, its physical and mental consequences would be far more.^15^ However, one question that needs to be asked is whether victims of intimate partner violence would experience self-dehumanization. Furthermore, far too little attention has been paid to the experience of being dehumanized by these women.

**Current study**

Although the experience of being dehumanized and dehumanizing the perpetrators can have devastating effects on the cognitions and emotions of the victims,^16^ little is known about experiencing dehumanization in victims of intimate partner violence. Apart from Bastain and Haslam, there is a general lack of research on the experience of being dehumanized by the victims. On the other hand, previous studies have investigated dehumanization in the context of sexual violence against women, but physical and psychological violence has been neglected so far. Furthermore, all the previously mentioned studies have serious limitations; for example, sexual violence has not been investigated among real victims in these studies.^9,17,18^ Thus, by employing a qualitative method, we attempt to illuminate the role of dehumanization and the experience of being dehumanized in psychological, physical, and sexual intimate partner violence.

## Methods 

**Participants**

Sampling in this study is conducted in two phases. In the first phase, participants were recruited from different areas of Tehran, Iran, across places such as parks and mosques through a convenience sampling method. Eligible women, who matched the selection criteria of experiencing intimate partner violence, were identified by the Revised Conflict Tactics Scale (CTS2) results. This scale is used to screen and identify victims of intimate partner violence. The initial sample consisted of 130 women, 60 of them, whose score in each of the subscales of the CTS2 scale was above the 25th percentile and had a score higher than 15, were selected. In the second phase, we used theoretical sampling, in which we interviewed these women purposively until we reached theoretical saturation with 10 participants.

**Study Procedures**

The procedure consists of in-depth interviews with female intimate partner violence victims selected by the CTS2 questionnaire. The data was recorded on a digital audio recorder and transcribed. The first interview was analyzed using Strauss and Corbin’s^19^ grounded theory method. The analysis consists of three levels of coding: open coding, axial coding, and selective coding. Three researchers were in the analysis group, and the codes and their relationships were checked repeatedly within the group. Interviews continued until the theoretical saturation of data had been made when no new information or categories were revealed as more data were collected.

**The Measures**

**revised conflict tactics scale CTS2.**The CTS2^20^ is a 78-item self-report measure that assesses five tactics by which conflict can be resolved in a marital, cohabiting, or dating relationship. It includes perpetrator and victim subscales. Each consists of six items for negotiation, eight for psychological aggression, twelve for physical assault, seven for sexual coercion, and six for injury. Each item is rated on an 8-point scale. The reliability and validity of the scale were studied by the authors in 1996 and showed that all five subscales were consistent and related to the theory.

In this study, the Persian form^21^ was used. Panaghi et al. showed that the Persian form has appropriate validity and reliability for the Iranian sample and had acceptable convergent and divergent validity compared with its subscales. The subscales had good internal consistency (Cronbach’s a=.66 to .86). 

**In-depth Interview. **Different authors have measured dehumanization in a variety of ways. Each has its advantages and drawbacks. This study used in-depth semi-structured interviews to identify the role of dehumanization and the experience of being dehumanized in intimate partner violence. The participants were asked about their emotions, thoughts, and behaviors encountering intimate partner violence. There were two questions in which we directly asked the interviewees about dehumanization and self-dehumanization, and the other questions explored this phenomenon in a subtler form in their behaviors, their thoughts and emotions about self, their partners, and their perception about their partners’ behaviors, thoughts, and emotions about them. Each interview lasted between 30 to 60 minutes. Some of the interview questions are listed below:

- What did you feel about yourself after experiencing intimate partner violence?

- While your husband battered you, what did you think he thought of you?

- What were your feelings about your husband after experiencing violence?

- Did you ever perceive yourself or your husband as less than human? Could you explain your experience?

- Do you ever think that your husband thinks you are less than human? 

**Data analysis:**

In this study, data analysis was based on Strauss and Corbin’s^19^ method using three levels of coding: open coding, axial coding, and selective coding. In open coding, the researcher creates small units of meanings and labels them. The next level is axial coding which involves grouping and labeling units from the previous phase into broader categories and identifying their relationships. The final stage, selective coding, involves integrating categories and their connections to identify a core category that applies to all categories and integrating the information into an explanatory model using diagrams. 

**Validity and Reliability:**

Lincoln and Guba^22^ presented a method to assess the reliability and validity of qualitative research. According to this approach, Authenticity and Trustworthiness in qualitative research replaced reliability and validity in quantitative research, indicating how thorough the researchers have been in the research process.

**The authenticity of Research. **A study has accuracy when it uses appropriate strategies to report the actual views of participants. In this study, we have tried to gather unbiased data from the interviews to indicate the participants’ real perspectives.

**Trustworthiness of research. **To confirm the reliability of the research, the following should be evaluated: 1. Credibility, 2. Transferability, 3. Dependability, 4. Confirmability.

*Credibility. *To enhance the credibility of the data, several triangulation methods were used. Data triangulation in this study was done using different groups of victims of violence (victims of psychological violence, sexual violence, or all three types of violence). Investigator triangulation was obtained in collaboration with researchers in the study. In this way, the data were periodically checked by the supervisors. 

*Transferability. *Connection with the scientific background of the study can be the basis for transferability. Furthermore, a detailed description of the procedure helps the transferability. In this study, we described the sampling and coding procedure in detail, and we described the scientific background of the study in the introduction and discussion sections.

*Dependability. *One way to achieve dependability is an audit. To achieve the audit, all actions of researchers from the beginning to the completion of the analysis were recorded to validate the entire process. We recorded the interviews and checked all research procedures with one of the professors to avoid researcher bias.

*Confirmability. *To show a study’s confirmability, it must demonstrate how the information is connected to its resources, and results and interpretations are directly extracted from the raw data. In this study, all the coding procedure from open coding, axial coding, and selective coding was provided in detail to the supervisors, and we used the audit to obtain confirmability.

## Results

**Demographic Characteristics of the Sample:**

The participants in this research were ten female victims of intimate partner violence. The demographic characteristics of the participants are demonstrated in Table 1 . Five participants were housewives; the rest were students, employees, or self-employed. Half of their husbands were employees, three were self-employed, one was a lawyer, and one was a teacher. Seven participants had bachelor’s degrees, two had high school diplomas, and one had an associate degree. All of their husbands had a bachelor’s degree except one that had dropped high school. One participant had a higher education than her husband, and three had a lower education. The rest of the participants had the same level of education as their husbands.

**Table 1:** Demographic characteristics of the participants and their partners.

Variables	Mean	Standard Deviation	Min. Age	Max. Age
**Participant’s age**	34.5	5.16	25	41
**Husbands’ age**	39.2	8.37	25	53
**Age differences**	4.7	5	0	13
**Years of being married**	12.2	7.98	1	25

Through open coding, 25 initial codes were identified; these codes were organized through open and axial coding into ten categories, some of which had sub-categories. These categories and their relations with each other are presented in great detail in Table 2. 

**Table 2:** Categories and Sub-categories emerged from open ad axial coding.

The role of dehumanization preceding Intimate partner vio-lence	The role of dehumanization following intimate partner vio-lence
**Attitudes of society toward women**	Intimate Partner Violence
	-Physical violence
	-Psychological violence
	-Sexual violence
**What men thought of their wives from their perspective**	Women’s Emotions:
**-Women’s low cognitive ability**	-Inferiority
**-Women being worthless**	-Hatred
	-Anger
**Strategies used by the victims in response to their husband’s violence**	Women’s Cognitions:
**-Active strategies**	-Women’s low cognitive ability in their husbands’ perspective
**-Attempting to leave the home**	-Women being worthless in their husbands’ perspective
**-Retaliating the violent husband**	-Being emotionally cold
**-Passive strategies**	-Being powerless
**-Regret of marriage**	-Illogicality of the husband
**-Cursing and wishing for the husband’s death**	-Husbands’ lack of understanding
**Thoughts associated with death and suicide**	-Husband worthlessness
	-Husbands’ impulsivity
**Dehumanization of women**	Experiencing Dehumanization:
	-Self-dehumanization
	-Dehumanization of husband
	-Experiencing being dehumanized
**Domestic violence:**	Strategies used by the victims in response to their husband’s violence
**-Physical violence**	-Active strategies
**-Psychological violence**	-Attempting to leave the home
**-Sexual violence**	-Retaliating the violent husband
	-Passive strategies
	-Regret of marriage
	-Cursing and wishing for the husband’s death
	-Thoughts associated with death and suicide
**Experiencing Dehumanization:**	Exacerbating dehumanization of women
**-Self-dehumanization**	
**-Dehumanization of husband**	
**-Experience of being dehumanized**	
	Aggravating factors:
	-Being pregnant
	-Presence of others

Through selective coding, the relations between the codes were identified, and it was revealed that the role of dehumanization in intimate partner violence in their perspectives could be demonstrated in two models and a cycle. The first model shows dehumanization preceding the occurrence of intimate partner violence, and the second demonstrates dehumanization following its occurrence. Each of these models involves the central phenomenon, the causal conditions, the intervening conditions, the contextual conditions, the action and interaction strategies, and the consequences. These two models form a cycle of dehumanization and intimate partner violence.

**The First Model**

**Figure 1:**
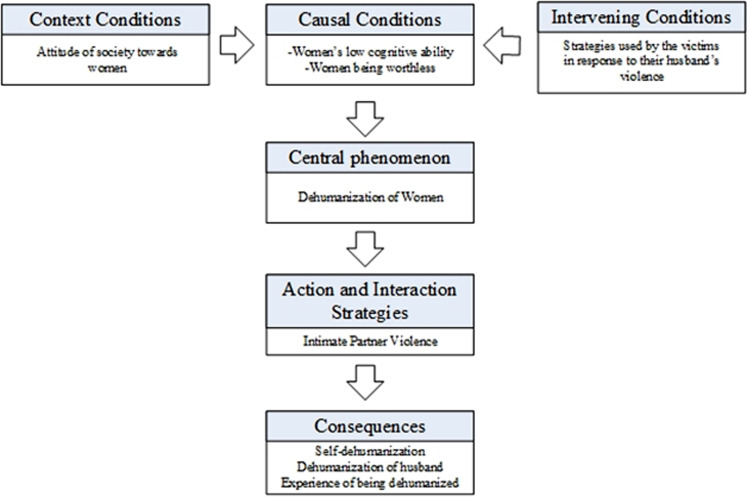
The role of dehumanization preceding intimate partner violence.

Figure 1 provides an overview of the first model that demonstrates how certain conditions lead to the dehumanization of women and, consequently, to intimate partner violence and how women perceive it and react to it. This model is explained in Strauss and Corbin’s^19^ grounded theory diagram, which involves central phenomenon, causal conditions, intervening conditions, contextual conditions, action and interaction strategies, and consequences. Each of these factors is explained in detail in the following paragraphs.

**Central phenomenon. **In the current model, the central phenomenon is the dehumanization of women. In this model, dehumanization can occur in animalistic and mechanistic forms. In this regard, one individual stated that” My husband sees me like an animal. I see him as an animal either. If he was a human, he could not do that to me, “and another commented, “My husband was a boxer, it was as if I was a boxing bag for him. He never considers me as a living thing”. One participant explained: “when we don’t fight, he says that a person only hits an animal, but when there is a fight, he hits me again,” one of the victims said, “He treated me like I am not a living thing,” similarly one participant asserted “That person looks at you like an object, like something worthless.”

**Causal conditions. **From these women’s viewpoint, “women’s low cognitive ability” and “worthlessness of women” in their husband’s perspective are the causal conditions that lead to dehumanization. In this study, men’s thoughts about their wives had two sub-categories: Women’s low cognitive ability and Women’s worthlessness. Some quotes support the former sub-category; for example, one participant stated, “If he valued me and saw me as a human being, he would not have done this,” one woman said, “My role in this house was being a maid and a babysitter. No one values me here,” and another explained “My husband is very obsessive about his personal belongings, no one dares to touch them at all. I wish I was like his belongings and valued like his car or T.V.”

Some other quotes supported the latter sub-category. In this regard, some participants talked about their conversations with their husbands; they heard things like: “You can not understand what I am saying,” “A two-year-old child is smarter than you. Your understanding is less than a child,” or “The wall understands me, but you don’t. If I had talked to a stone, It would understand me.” In response to the question:” What do you think your husband believes about you or other women?” a woman said: “He says to me, you are an idiot, you are not even a human, you are an animal, you cannot understand me.”

**Intervening conditions. **In this model, the intervening conditions involve the strategies used by the victims of intimate partner violence in response to their husband’s violence. As the next model explains, women use passive and active strategies in response to their husbands’ violence. According to the common belief of these women, these strategies could lead to more dehumanizing beliefs in their husbands. These strategies are explained in detail in the next model.

**Contextual conditions. **In this model, contextual conditions include certain attitudes of society towards women. According to the victims of intimate partner violence, society’s attitude of women’s inferiority might increase dehumanization of them. Some of these attitudes of inferiority are as follows: Women should not work, women should not be independent, women are intellectually weak, and women are worthless. In this regard, some victims said their husbands were affected by these societal attitudes and believed in them. For example, some of them asserted, “He believed that women should not be independent, and he didn’t let me have a job, and now most of my problems are due to this” another woman said, “In his opinion, women are stupid. For example, if a woman is in front of him while driving, he will insult her.” These perspectives, alongside other thoughts related to “low cognitive ability” and “worthlessness,” explained in the causal conditions section, can intensify women’s dehumanization in intimate contexts. 

**Action and interaction strategies. **According to the participants, dehumanization leads to all kinds of intimate partner violence. In the present research, IPV was observed in three forms: psychological, physical, and sexual. 

Some participants reported psychological violence and said: “I felt that I was being ignored,” or “Sometimes he doesn’t even answer my questions, he just nods his head,” or “If he wants to answer me, he will shout it. He doesn’t want to solve our problems like a logical person,” and “When he yells at me and insults me, I feel crushed.” Victims who reported physical violence mainly explain it like this: “He beats me terribly without thinking about the outcomes,” and “He beats me and doesn’t think what will happen next.” Some other participants reported sexual violence, examples of their description of their experiences were: “He wanted sex without caring about my desires,” and “When he sees that I don’t want to have sex with him, he should at least try a little... not doing what he wants, like animals that don’t have any foreplay. At least try to prepare me. He treated me like an animal.”

**Consequence. **The consequences of violence in an intimate relationship could lead to self-dehumanization and the experience of being dehumanized. It also leads to the dehumanization of the husband as the perpetrator of the violence. 

In this regard, some victims of intimate partner violence, in response to the question about how they felt about their husbands and whether they see them as less than human, pointed out partner dehumanization and stated: “He doesn’t think at all about what he says, it is like that he says some recorded things like a robot,” or “If he were a human, he would understand what I am saying, but now he just repeats himself like a machine,” “He had no value for me at all. In my opinion he was not different from this table. I had no feelings for him,” and “In my opinion, in the relationship between a husband and a wife, feelings come first. You have a relationship with an animal if this feeling is ignored.”

Some other answers of the victims about their feelings about themselves indicate self-dehumanization, for example: “It is as if I have no control over myself. I am a tool in his hands,” or “I felt that I had to obey him like an emotionless doll,” and “When he yells at me, I don’t move, I don’t react just like a statue.”

**The second model**

**Figure 2:**
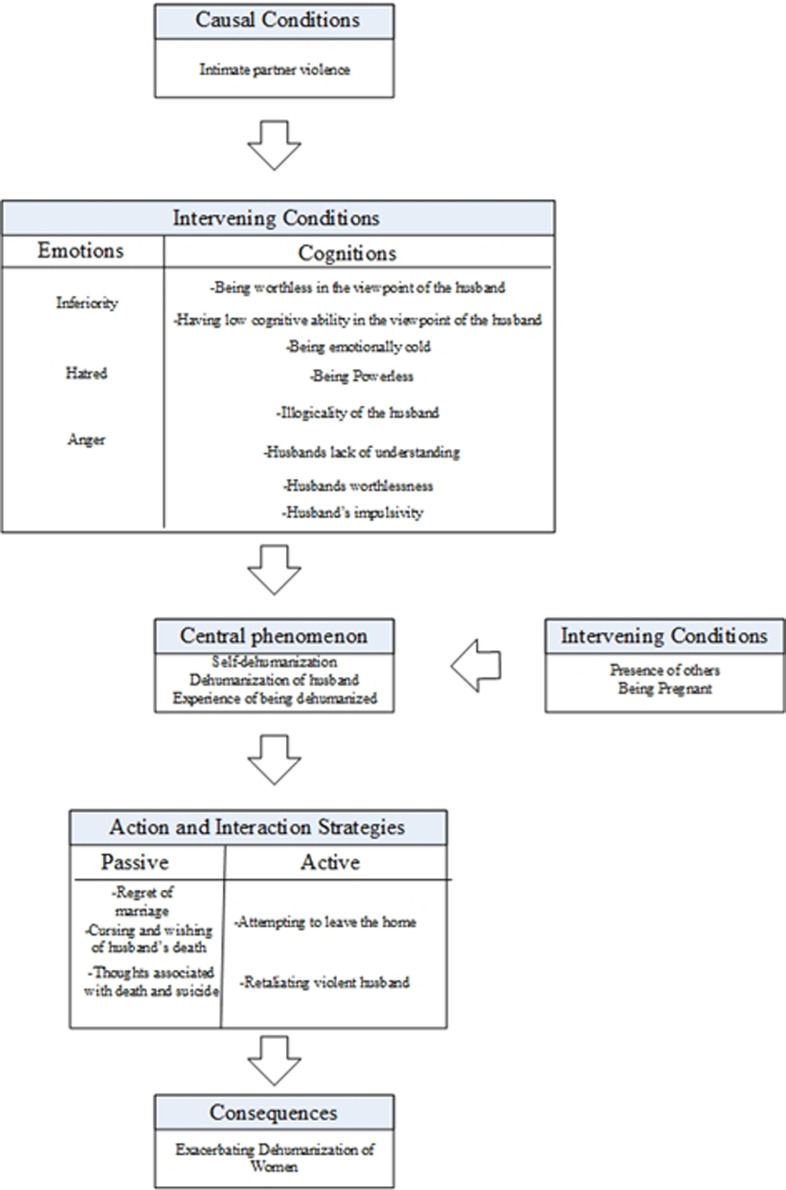
The role of dehumanization following intimate partner violence.

Dehumanization from women’s perspective after experiencing intimate partner violence is demonstrated in the second model (see Figure 2). This model is also explained in Strauss and Corbin’s ^19^ grounded theory diagram, which involves central phenomenon, causal conditions, intervening conditions, contextual conditions, action and interaction strategies, and consequences. 

**The central phenomenon. **The central phenomenon in this model is dehumanization. Dehumanization here is seen as self-dehumanization, dehumanization of the husband, and the experience of being dehumanized. In this model, dehumanization can also occur in animalistic and mechanistic forms. 

**Causal conditions. **In this model, the main causal conditions or the conditions that lead to the occurrence of the central phenomenon include intimate partner violence. Intimate partner violence in this study includes psychological, physical, and sexual violence that leads to dehumanizing the husband, self, and experience of being dehumanized. The majority of those who responded to this item experienced this phenomenon as “rampage,” “profanity,” “fear,” and “prevention of independent activity.” Physical violence in these women was seen as being beaten by their intimate partners, and sexual violence was seen in forced sexual intercourse and unusual sexual relations.

**Intervening conditions. **Intervening conditions in this model involve the emotions and cognitions of abused women. Intervening cognitions involve “being worthless in the viewpoint of the husband,” “having low cognitive ability in the viewpoint of the husband,” “being emotionally cold,” “being powerless,” “illogicality of husband,” “husband’s lack of understanding, “husband’s worthlessness” and “husband’s impulsivity.” The intervention emotions also include the feeling of “inferiority,” “hatred” and “anger.”

Participants talked about these cognitions and emotions throughout the interview; some examples of them are reported here respectively: “In the beginning, I had a lot of feelings for him, or if we fought, I would be upset. But now I don’t have any feelings anymore. I see myself cold and mechanical,” “I felt that I had no control over myself. Because I didn’t want it, it was only because of his wish. If he said yes, I should have said yes. If he said no, I should have said no,” “My husband never had any logic, he just ranting and raving, his actions had no logic or reason,” “I feel that my husband does not understand me at all, he only speaks for himself, like a tape recorder” “He doesn’t matter to me anymore. A few days ago, when he told me about his accident, I said that it had nothing to do with me,” “I feel like my husband is treating me like an animal without thinking,” “I felt humiliated at that moment. When I compared myself with others, I felt inferior,” “After that, I hated him,” and “When my husband beats me, I feel angry.”

A common view among interviewees was that the perception of being emotionally cold, powerlessness, and feeling inferior leads to self-dehumanization. Perceiving the husband as an illogical person, the low cognitive abilities of the husband, being worthless and impulsive, and feelings of anger and hatred are the intervening factors in dehumanizing the husband.

**Context conditions. **Female victims of intimate partner violence believe that pregnancy and the presence of others at the time of violence are the major contexts in which dehumanization occurs. Some interviewees argued that the most compelling reason they experienced dehumanization was their pregnancy at the time of violence. Others stated that the presence of other people at the time of violence was the contextual reason for this experience.

For example, in response to the question, “when were these feelings more than other times?” one interviewee said, “One time I remember well was when I was pregnant with my first child. I was only 19. He severely battered me to bleeding. I thought the baby would die,” and the other stated: “What made it stick in my mind is that I was pregnant at that time, my husband told me that I did that to kill the baby.” Another interviewee said: “I remember a time; he slapped me in front of my brother-in-law. It was hard for me to be beaten in front of him,” and another stated:” The time he beat me in front of the guests was the worst.”

**Action and interaction strategies. **Female victims of violence use a variety of strategies in response to experiencing dehumanization. These strategies can be categorized into passive and active strategies. Active strategies include “abandoning home” and “retaliating against husband’s violence.” Passive strategies include “regret of marriage,” “cursing and wishing for husband’s death,” and “thoughts associated with death and suicide.”

In this regard, an interviewee stated, “I felt that I was wrong; he was not the person I expected, and I have regretted all these years.” A participant said, “I asked God to do something so that he won’t return back, something happens to him, or he dies; I wanted to kill him. I was thinking of what to do to kill him.” Another participant argued, “I wish that he dies or I die,” another participant stated, “I decided to commit suicide several times, but I did not dare.” On the other hand, some participants expressed active strategies. One participant reported, “I decided to behave like him. If he insults me, I will do the same “. Another interviewee stated, “many times I decided to escape. Even once I gathered my things to go to Mashhad. I was going there to be a shrine server”. With a closer look at these strategies, it could be argued that they are hidden and indirect violence and, in some cases, open and direct aggressions.

**Consequences. **The consequence of strategies used by female victims of violence exacerbates women’s dehumanization. In other words, when women use these strategies, the idea of their husbands that women cannot understand them is reinforced. There was a sense amongst interviewees that the husband’s feeling of not being understood by women, alongside other contextual conditions, could lead to intensified dehumanization of women and intimate partner violence. 

 For example, a violent husband would think that his ideas and thought cannot be understood by his wife when she retaliates against her husband or curses him. These ideas can lead the husband to dehumanize her more; therefore, more intimate partner violence would occur. When the woman experiences more violence, it will lead to more partner and self-dehumanization, and subsequently active and passive strategies again, forming a cycle between violence and dehumanization.

**The cycle of domestic violence and dehumanization**

**Figure 3:**
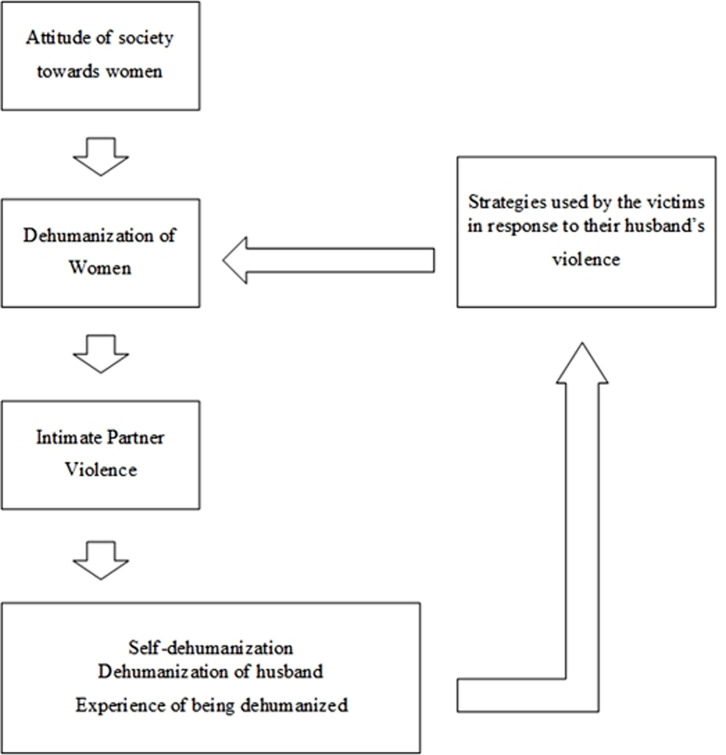
Combination of two models of dehumanization in intimate partner violence.

According to the models discussed, dehumanization and violence seem closely associated. Dehumanization of women, which is mainly affected by society’s attitudes toward women, leads to intimate partner violence, self-dehumanization, dehumanizing the husband, and the experience of being dehumanized. As mentioned in the two previous models, experiencing these three types of dehumanization by women leads to strategies that might intensify the women’s dehumanization by their husbands. Therefore, it could be concluded that integrating the abovementioned models can form a vicious cycle (see Figure 3).

Together these results provide important insights into the role of dehumanization and the experience of being dehumanized in intimate partner violence and its consequences. What is interesting about this data is that dehumanization can be both a consequence of violence and a causal condition. Therefore, it can be concluded that the relationship between dehumanization and violence is bilateral rather than linear. 

## Discussion

**Dehumanization in the context of intimate partner violence**

The current study showed that victims of intimate partner violence think they are dehumanized by their spouses, eventually leading to intimate partner violence. Many studies have demonstrated the relationship between psychological and physical violence and dehumanization. In this regard, Brennan ^8^ affirmed that dehumanization is associated with verbal violence, regarded as psychological violence. Moreover, several studies have recognized dehumanization in the context of sexual violence against women.^9,23,24^

Data analysis revealed that dehumanization could also be a consequence of intimate partner violence. According to the victims of this study, women had experienced dehumanization by their husbands, dehumanize their husbands, and are vulnerable to self-dehumanization. There are several possible explanations for this result. It could be stated that the feeling of being powerless in these women is caused by being controlled by their spouses. Results from Yang and Jin^25^ indicated that people with the least power in society dehumanize themselves and those responsible for their current situation. In our study, feeling powerless in women is consistent with these findings. Moller and Deci^1^ also affirmed that the feeling of being controlled by others leads to mechanistic self-dehumanization and dehumanization of the perpetrator.

On the other hand, self-dehumanization could be regarded as “contempt toward self.” In a study by Sadeghi and Mazaheri,^26^ “contempt toward self” in distressed couples among the Iranian population can be attributed to the autonomy of the individual in protecting himself/herself against the authoritarian power of his/her spouse. It can also be the condition where one of the couples wants to express his/her regret for being in this relationship. 

**Intervening and contextual conditions of dehumanization in the context of intimate partner violence**

It was demonstrated in this study that experiencing dehumanization could be intensified during pregnancies or in the existence of other people. These results are consistent with other studies and suggest that pregnant women are more likely to be the target of dehumanization or violence. In a study by Goldenberg and Goplen,^27^ it was illustrated that pregnant women are more likely to be the target of offensive behaviors and animalistic dehumanization than non-pregnant women. Furthermore, it could be stated that with being a target of violence in front of other people, the feeling of inferiority intensifies in women. In this regard, it was demonstrated in studies by Haslam,^2^ Bastian and Haslam,^12^ and Esses, Veenvliet^28^ that the feeling of inferiority was highly correlated with dehumanization. Therefore, the possibility of dehumanization will be higher in such situations.

According to our findings, another main factor underlying experiencing dehumanization is gender stereotypes and viewing women as an inferior social group. This result may be explained based on the feminist perspective and patriarchal ideology.^29,30^ Another possible explanation for this might be that, according to the ecological systems theory,^31^ structures, cultural systems, values, and attitudes affect a more comprehensive range of the lower levels at the ecosystem level. In this regard, Heise ^32^ marked that believing in the dominance of men over women, the relationship between masculinity and violence, strict gender roles, and interpersonal acceptance of violence could be associated with increased violence in lower levels or, in other words, the family level. On the other hand, the impacts of social and cultural viewpoints on dehumanization have been evaluated in several studies. In this regard, Harris and Fiske^33^ affirmed that a group that is considered socially inferior is more prone to dehumanization. 

**Consequences of dehumanization in the context of intimate partner violence**

Dehumanization in victims of intimate partner violence could be associated with different emotions. It was stated earlier that dehumanization led to a sense of inferiority in women, which is in congruence with the dual model of Haslam.^2^ According to this model, animalistic dehumanization is associated with a sense of inferiority. It seems possible that these results are due to the feeling of worthlessness caused by the dehumanization. According to the results of the present study, dehumanizing the husband is associated with feelings of hatred and anger, which has also been indicated in the studies by Haslam,^2^ Hodson and Costello,^34^ and Harris and Fiske.^33^ In this regard, Jack and Dawson ^35^ affirmed that animalistic dehumanization raises the victims’ adverse emotional reactions (such as hatred). The anger toward the dehumanizer was also observed in the studies by Haslam^2^ and Bastian and Haslam.^12^ In this regard, it was demonstrated in a study by Fischer and Roseman^36^ that reduced intimacy between the spouses and being controlled by the husbands caused a feeling of hatred in women. In the current research, being controlled by the husband, which is one of the factors of dehumanization, was regarded as one of the causes of hatred and anger in the dehumanized women.

The current research indicated that women used strategies against themselves or their spouses in response to their husbands’ dehumanization and self-dehumanization, divided into two active and passive categories. Active strategies included suicidal and self-destructive thoughts, violence toward the spouse, or leaving their homes. On the other hand, passive strategies include regretting their marriage, hatred, and wishing for the death of their husbands. A study by Buss^37^ showed that passive violence is a strategy against people with higher social levels. In another study, greater use of direct violence was observed in men, whereas greater use of indirect violence was found in women.^38^

Furthermore, suicidal thoughts are regarded as self-violence based on the catharsis theory.^39^ In addition, suicide is known as a strategy to escape from painful self-consciousness in the theory of Baumeister.^40^ It could be stated that this self-consciousness is, in fact, self-dehumanization or the realization of being dehumanized by the spouse and feeling of inferiority. 

**A cycle of dehumanization and intimate partner violence**

The most interesting finding of this study was that dehumanization and violence form a vicious cycle. Dehumanization of women has led to violence against them, which will be associated with the dehumanization of the spouse, self-dehumanization, and the experience of being dehumanized. Responding to such behaviors, women use active or passive-aggressive strategies, which lead to intensified dehumanization by their spouses. In a study by Bastian and Haslam, ^13^ it was noted that a cycle of dehumanization and violence could exist, but no specific studies have demonstrated this cycle and its factors.

## Conclusion

This study aimed to determine the role of dehumanization and the experience of being dehumanized in intimate partner violence. Although the current research is based on a small sample of participants, the findings confirm previous findings and contribute additional evidence that suggests dehumanization could occur in two forms, with three targets in families and intimate relationships, which leads to aggressive behavior.

One of the more significant findings from this study is that dehumanization could invoke negative cognitions, emotions, and strategies. The second significant finding was that dehumanization involves a cycle of intimate partner violence. This study has gone some way toward enhancing our understanding of dehumanization in close relationships. More research on this topic needs to be undertaken before the association between dehumanization, and intimate partner violence is more clearly understood. Furthermore, the dehumanization of the perpetrator partner in victims of intimate partner violence should be explored in more detail in future studies to clarify its features and consequences, especially from the perpetrators’ perspective. 

**Acknowledgments**

We would like to express our appreciation to Professor Nick Haslam for his generous support.
